# Source and Impact of the EGF Family of Ligands on Intestinal Stem Cells

**DOI:** 10.3389/fcell.2021.685665

**Published:** 2021-07-19

**Authors:** Helen E. Abud, Wing Hei Chan, Thierry Jardé

**Affiliations:** ^1^Department of Anatomy and Developmental Biology, Monash University, Clayton, VIC, Australia; ^2^Development and Stem Cells Program, Monash Biomedicine Discovery Institute, Clayton, VIC, Australia

**Keywords:** Epidermal Growth Factor, neuregulin 1, intestinal stem cells, signaling, niche, ErbB, tissue regeneration, organoids

## Abstract

Epidermal Growth Factor (EGF) has long been known for its role in promoting proliferation of intestinal epithelial cells. EGF is produced by epithelial niche cells at the base of crypts *in vivo* and is routinely added to the culture medium to support the growth of intestinal organoids *ex vivo*. The recent identification of diverse stromal cell populations that reside underneath intestinal crypts has enabled the characterization of key growth factor cues supplied by these cells. The nature of these signals and how they are delivered to drive intestinal epithelial development, daily homeostasis and tissue regeneration following injury are being investigated. It is clear that aside from EGF, other ligands of the family, including Neuregulin 1 (NRG1), have distinct roles in supporting the function of intestinal stem cells through the ErbB pathway.

## Introduction

A balance between cell proliferation and differentiation is exquisitely controlled in the intestinal epithelium throughout life (Beumer and Clevers, 2021). This is coordinated by a system of cues from surrounding niche cells that includes Paneth cells in the epithelium (Sato et al., 2011), diverse populations of stromal cells (Hageman et al., 2020; McCarthy et al., 2020a; Sphyris et al., 2021), enteric neural cells (Van Landeghem et al., 2011; Talbot et al., 2020) and macrophages (De Schepper et al., 2018; Sehgal et al., 2018). These signals act on the epithelium to modulate stem cell function and cell fate acquisition in progenitor cells (Tetteh et al., 2016). This complex array of cellular inputs has the ability to support the enormous expansion of the intestinal tract during development (Chin et al., 2017) and the strong proliferative response occurring during tissue repair following damage to ensure integrity of the epithelium (Hageman et al., 2020). Maintenance of the barrier and adequate tissue function is vital to prevent systemic infection from enteric pathogens and adequate digestion and nutrient absorption. Stem cells, which reside in the base of intestinal crypts, either self-renew or generate transit-amplifying progenitor cells that ultimately differentiate and generate the diversity of secretory and absorptive differentiated cell types required for a functional epithelium (Beumer and Clevers, 2021). Interplay of key signals from the WNT, Notch, Epidermal Growth Factor (EGF), and Bone Morphogenetic Protein (BMP) signaling pathways regulate the survival, self-renewal and differentiation of these cells to ensure a balance of cell types (Holik et al., 2013; Horvay and Abud, 2013; Tian et al., 2015; McCarthy et al., 2020b; Beumer and Clevers, 2021). The exact mechanisms of how signals are produced, what cell types secrete and receive signals and how tissues respond to promote the process of regeneration are being investigated. Some of the cell types that secrete molecules that either augment or inhibit WNT and BMP signaling have been described. EGF is present in Paneth cells and has primarily been shown to influence proliferation (Abud et al., 2005; Basak et al., 2017), but it is becoming increasingly clear that other ligands from this family can also influence the diversity of cells within the epithelium (Jardé et al., 2020; Holloway et al., 2021). Whether these ligands have distinct or functionally redundant activities and how signals influence the epithelium in different contexts is still being investigated. In this review, we discuss current evidence on the cellular source and role of ligands from the EGF family and how they interact with receptors in the epithelium to influence cellular proliferation, stem cell identity and lineage differentiation.

## The EGF Family of Receptors and Ligands

**FIGURE 1:**
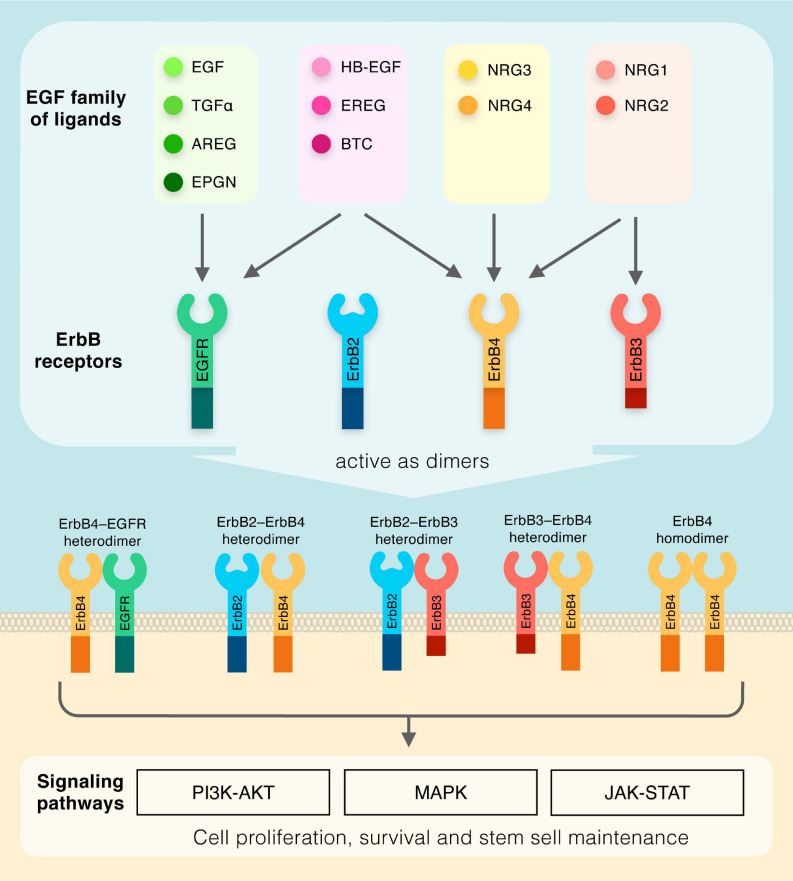
Diagram outlining the members of the EGF family of ligands and their respective receptors. Dimeric receptors activate downstream PI3K-AKT, MAPK, and JAK-STAT signaling pathways.

The EGF family of ligands includes eleven structurally related proteins, namely EGF, transforming growth factor α (TGF-α), amphiregulin (AREG), epigen (EPGN), heparin-binding EGF-like growth factor (HB-EGF), epiregulin (EREG), betacellulin (BTC), and the neuregulins (NRG1-4) (Figure 1). These molecules have in common similar EGF-like motifs, and, due to their membrane-anchored nature, can act in a juxtacrine manner between two neighboring cells, or, in an autocrine/paracrine mode via proteolytic cleavage of the external EGF-like domain, which results in its release in the extracellular compartment (Singh and Harris, 2005; Rayego-Mateos et al., 2018). The EGF-like protein drives cellular signal transduction through the ErbB subclass of the Receptor Tyrosine Kinase superfamily, which consists of four members EGFR (also known as ErbB1), ErbB2, ErbB3, and ErbB4 (Downward et al., 1984; Schechter et al., 1984; Semba et al., 1985; Kraus et al., 1989; Plowman et al., 1993). The EGF family of ligands can be classified into four sub-groups based on distinct receptor binding specificities: (1) the ligands which recognize ErbB1 only (EGF, TGFα, AREG, and EPGN); (2) the ligands binding to both ErbB1 and ErbB4 (HB-EGF, EREG, and BTC); (3) the ligands which are specific for both ErbB3 and ErbB4 (NRG1 and NRG2); and (4) the ligands activating ErbB4 only (NRG3 and NRG4) (Figure 1). It should be noted that no ligands have been identified for ErbB2 to date. Nonetheless, all ErbB receptors contain an extracellular ligand binding site, a single membrane spanning region and a cytoplasmic tyrosine-kinase-containing domain (Lemmon et al., 2014). Upon ligand-induced conformational change, ErbB receptors form homodimers or heterodimers (Figure 1), which activates the intrinsic kinase domain, resulting in the phosphorylation of specific tyrosine residues within the cytoplasmic tails (Lemmon et al., 2014). Phosphorylated residues serve as docking sites for a range of molecules and regulatory proteins involved in various cascades of intracellular signaling, including MAPK, PI3K-AKT, and JAK-STAT (Singh and Harris, 2005; Iwakura and Nawa, 2013). These complex signaling routes regulate many key cellular functions, including cell proliferation, cell death and stem cell maintenance, which are essential for numerous body systems.

## Location and Function of Signals and Receptors During Mammalian Homeostasis

**FIGURE 2:**
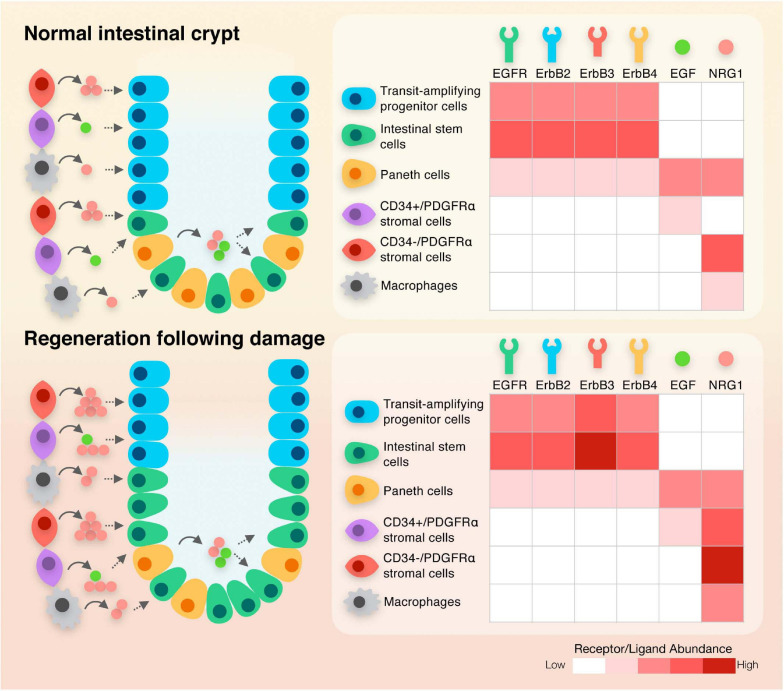
Schematic diagram depicting the expression of ligands and receptors within the different cellular compartments of the intestinal crypt during normal homeostasis and following regeneration.

In mice and humans, Paneth cells are localized within the epithelium adjacent to intestinal stem cells in the base of crypts. These specialized cells secrete WNT3, EGF and NRG1 during intestinal homeostasis (Sato et al., 2011; Jardé et al., 2020; Figure 2). Although initially identified as a key constituent of the niche, it is clear that when Paneth cells are depleted *in vivo*, other cellular sources can compensate for this loss, resulting in the maintenance of an intact epithelium (Durand et al., 2012; Farin et al., 2012). The stromal compartment of the adult intestinal tract contains sub-populations of cells including FOXL1+ telocytes that secrete WNT2B and the WNT signaling potentiator RSPO3 (Aoki et al., 2016; Shoshkes-Carmel et al., 2018) and PDGFRα+ cells (Greicius et al., 2018; McCarthy et al., 2020b) that also secrete WNT2B and RSPO3. Mesenchymal cells marked by GLI1 also secrete WNT in the colon (Degirmenci et al., 2018). Single cell sequencing has revealed distinct sub-populations that express different levels of PDGFRα, with those expressing higher levels being localized to the villi, and CD34+ PDGFRα^low^ cells residing at the base of crypts, where they express *Grem1* and antagonize BMP signaling. These cells described as trophocytes are capable of fully supporting the growth of organoids *ex vivo* (McCarthy et al., 2020b). A similar sub-population of fibroblasts associated with crypts was also recently identified in the colon (Brugger et al., 2020). MAP3K2 expressing cells also reside at the base of crypts where they secrete RSPO1 (Wu et al., 2021). Macrophages identified by expression of CD11b and CSF1R are also closely associated with crypts and when depleted, loss of intestinal stem cells are observed (De Schepper et al., 2018; Sehgal et al., 2018). A unifying global expression analysis of the EGF family of ligands in both epithelial and mesenchymal intestinal niche sub-populations during intestinal homeostasis is currently missing. Re-analysis of recently published single cell RNA sequencing datasets would help understanding the complex ligand dynamics in the intestinal tract (Kinchen et al., 2018; Kim et al., 2020; Busslinger et al., 2021; Yu et al., 2021). Studies, which focused on individual ligands, have shown extremely low levels of EGF can be detected in mesenchymal cell populations, while NRG1 is expressed at relatively high levels in stromal cells, including PDGFRα+ cells, and is observed in macrophages (Jardé et al., 2020). Both EGF and NRG1-expressing cells are also found in the developing human intestinal tract, with EGF localized in the epithelial villus domain and NRG1 detected in PDGFRα+ cells within the subepithelial mesenchyme underlying crypts (Holloway et al., 2021; Yu et al., 2021). Other members of the NRG family of ligands display exclusive low expression in the mesenchyme (NRG2) or in the epithelium (NRG4) while NRG3 is absent in both compartments in adult intestinal tissues. AREG shares a similar expression pattern with NRG1, with an enrichment in subepithelial myofibroblasts and in rare F4/80+ macrophage-enriched cells but with limited expression in the epithelium (Inatomi et al., 2006; Shao and Sheng, 2010; Yang et al., 2017). EREG is also localized in an uncharacterized subpopulation of mesenchymal cells and is weakly expressed in the epithelium (Xian et al., 1999; Kallincos et al., 2000; Xia et al., 2003; Lee et al., 2004). In contrast, BTC, HB-EGF, and TGFα are enriched in the intestinal epithelium. The expression pattern of EPGN in both mesenchymal and epithelial compartments is currently unknown. Interestingly, EGFR/ERBB receptors are present within the epithelial cells of the epithelium (Figure 2). EGFR is enriched in stem and progenitor cells while ERBB2 and ERBB3 are detected throughout the crypt – villus axis (Suzuki et al., 2010; Jardé et al., 2020). Taken together, these data suggest a model where mesenchymal-secreted molecules, which includes NRG1, NRG2, AREG, and EREG, act on epithelial cells *via* a paracrine mechanism while epithelial-produced ligands such as EGF, BTC, HB-EGF, and TGFα regulate cellular function in an autocrine manner. It is important to note that the processes and signaling pathways regulating ligand production during intestinal homeostasis in these particular sub-types is not clearly defined. In addition, a comparative analysis of ligand-mediated downstream signaling pathways in normal intestinal cells is not available.

Numerous mouse knockout studies have investigated the functional roles of individual ligands (summarized in Table 1). There is likely to be some functional redundancy given triple knockout of three of the ligands EGF, AREG, and TGFα has little effect on the intestinal epithelium with animals being viable and fertile (Luetteke et al., 1999). Similarly, single knockout of the other EGF ligands does not affect embryonic development, with the exception of *Nrg1*, which results in aberrant heart and neural development leading to embryonic lethality (Meyer and Birchmeier, 1995; Erickson et al., 1997). Although knockout of EGF in mice produces little effect on the epithelium, the loss of NRG1 under homeostatic conditions produces a significant reduction in proliferative stem and progenitor cells (Jardé et al., 2020; Table 1). Loss of each individual ERBB receptor is lethal and produces severe defects with significant intestinal abnormalities observed (Gassmann et al., 1995; Lee et al., 1995; Miettinen et al., 1995; Threadgill et al., 1995; Erickson et al., 1997; Riethmacher et al., 1997). Genetic background has a considerable effect on the penetrance of phenotypes (see Table 1) with knockout of EGFR exhibiting variable phenotypes from peri-implantation lethality to viable animals with multi-organ defects (Sibilia and Wagner, 1995; Threadgill et al., 1995). Individual intestinal-specific knockout of ERBB receptors has also been reported to have minimal phenotypic effects during tissue homeostasis, suggesting some functional redundancy (Lee et al., 2009; Zhang et al., 2012; Almohazey et al., 2017; Srivatsa et al., 2017). However, double and/or triple knockout combinations that are intestinal-specific will be required to confirm this.

**TABLE 1:** Knockout mouse models of EGF family of ligands and receptors and their associated phenotypes.

**Ligand**	**Phenotype**	**Study**
*Egf*	Egf null mice are viable and fertile and display no overt phenotype, including in the gastrointestinal tract.	Luetteke et al., 1999
*Areg*	Areg KO mice are viable and fertile and display no overt phenotype, including in the gastrointestinal tract.	Luetteke et al., 1999
*Areg*	Loss of Areg significantly decreases the number of regenerating crypts following radiation-induced injury.	Shao and Sheng, 2010
*Tgf*α	Tgfα mutant mice are viable and fertile, but exhibit hair and eye defects.	Luetteke et al., 1993
*Egf/Areg/Tgf*α	Triple KO mice survive to maturity and display hair and eyes abnormalities consistent with single TGFα KO.	Luetteke et al., 1999
*Epgn*	Homozygous mutant mice are viable and fertile, and display no abnormal phenotype.	Dahlhoff et al., 2013
*HB-Egf*	KO mice are viable and fertile. Normal gastrointestinal tract architecture, but heart valve malformation.	Jackson et al., 2003
*HB-Egf*	Mutant mice are viable, but exhibit heart malformation.	Iwamoto et al., 2003
*Ereg*	Epiregulin null mice are morphologically normal and display no overt abnormal phenotype, including in the gut.	Lee et al., 2004
	However, KO mice display increased susceptibility to DSS-induced intestinal damage.	
*Ereg*	KO mice are viable, but display chronic dermatitis.	Shirasawa et al., 2004
*Btc*	Mutant mice are viable, fertile and display normal growth. No overt phenotype.	Jackson et al., 2003
*Nrg1*	Mutant animals die during development and display heart and nervous system aberrant phenotypes.	Meyer and Birchmeier, 1995
*Nrg1*	Embryonic lethality of mice deficient in Neuregulin Igl domain. Abnormal heart and cranial nerve development.	Kramer et al., 1996
*Nrg1*	Nrg1 null embryos die at E10.5 due to abnormal heart development.	Erickson et al., 1997
*Nrg1*	Inducible loss of Nrg1 in adults affects intestinal cell proliferation and stem cell maintenance during tissue homeostasis and regeneration.	Jardé et al., 2020
*Nrg2*	Nrg2 KO pups are viable but significantly smaller than their littermates. Analysis of major organs revealed no obvious changes.	Britto et al., 2004
*Nrg2*	Mutant mice are viable, but exhibit behavioral disorders.	Yan et al., 2018
*Nrg3*	KO mice are viable and fertile, but exhibit behavioral disorders.	Hayes et al., 2016
*Nrg4*	Nrg4 mutant mice are viable, but display metabolic disorders.	Wang et al., 2014

**Receptor**	**Phenotype**	**Study**

*Egfr*	Egfr KO on a CF-1 background results in peri-implantation death.	Threadgill et al., 1995
	On a 129/Sv background, homozygous mutants die at mid-gestation due to placental defects.	
	CD-1 mutants live for up to 3 weeks and show abnormalities in numerous tissues, including the gastrointestinal tract.	
*Egfr*	Embryonically lethal, but some mutant mice survive for up to 8 days after birth and display abnormal development, including in the gut.	Miettinen et al., 1995
*Egfr*	Egfr mutant fetuses on a 129/Sv background are retarded in growth and die at mid-gestation due to placental defects.	Sibilia and Wagner, 1995
	Some mice on a 129/Sv - C57BL/6 background survive until birth and to postnatal day 20 on a 129/Sv - C57BL/6 - MF1 background.	
	Newborn mutant mice display skin and lung phenotypes, but normal gastrointestinal tract.	
*Egfr (i)*	Mice harboring intestinal specific loss of Egfr are viable and display no obvious gut abnormalities.	Srivatsa et al., 2017
*Erbb2*	Erbb2 null embryos die before E11 due to abnormal cardiac and neural development.	Lee et al., 1995
*Erbb2*	Erbb2 KO embryos die on E10.5 and display cardiac and neural malfunction.	Erickson et al., 1997
*Erbb2 (i)*	Mice harboring intestinal specific loss of Erbb2 are viable and display no obvious gut abnormalities.	Zhang et al., 2012
	However, ErbB2 is required for tissue regeneration following DSS mediated injury.	
*Erbb3*	Erbb3 null embryos die at E10.5 due to neural defect. No developmental defects in epithelia of Erbb3 mutant embryos.	Riethmacher et al., 1997
*Erbb3*	Erbb3 loss is embryonically lethal at E13.5. Mice display cardiac, neural and gastrointestinal defects.	Erickson et al., 1997
*Erbb3*	Erbb3 KO results in embryonic lethality.	Lee et al., 2009
*Erbb3 (i)*	The intestinal epithelium of mice with intestine-specific genetic ablation of ErbB3 exhibits no cytological abnormalities.	Lee et al., 2009
	However, Erbb3 KO mice display more severe intestinal injury mediated by DSS.	
*Erbb3 (i)*	Deletion of intestinal epithelial Erbb3 in adult mice do not cause defects in architecture of the small intestine or colon.	Zhang et al., 2012
	However, Erbb3 is required for tissue regeneration following DSS-mediated injury.	
*Erbb3 (i)*	Intestinal epithelial Erbb3 KO causes early appearance of Paneth cells.	Almohazey et al., 2017
	Erbb3 KO mice are more sensitive to intestinal damage mediated by DSS.	
*Erbb4*	Erbb4 loss is embryonically lethal. Mice display cardiac and neural defects.	Gassmann et al., 1995
*Erbb4 (i)*	Deletion of intestinal epithelial Erbb4 in adult mice do not cause intestinal defects.	Almohazey et al., 2017

*(i) indicates intestinal specific deletion.*

## Role of the EGF Family of Ligands and Receptors During Regeneration Following Injury

The intestinal epithelium is a selective permeable barrier that permits uptake of nutrients from the luminal contents while forming a barrier against the toxic by-products of digestion and pathogenic bacteria (Beumer and Clevers, 2021). As the epithelial monolayer is exposed to an extremely harsh chemical and mechanical environment, it is highly vulnerable to damage. This is partially compensated for by the daily dynamic renewal of the epithelial layer, with differentiated cells being replaced every few days via the activity and neutral competition of a small population of stem cells (Snippert et al., 2010). Damage induced by pathogenic bacteria that primarily impacts the villi is rapidly repaired, but severe infection can also compromise the function of stem cells deep within crypts and result in more extensive damage (Mileto et al., 2020). Inflammation and treatments such as chemotherapy and radiotherapy also impact the integrity of cells within crypts. Recovery from these insults involves a repair and regeneration process that involves extensive remodeling of both cells within the epithelium and the surrounding niche cells (Hageman et al., 2020). Following injury, macrophages are recruited and secrete WNT ligands and IL6 (Taniguchi et al., 2015), innate lymphoid cells secrete IL22 (Aparicio-Domingo et al., 2015), stromal cells are remodeled (Kinchen et al., 2018) and there are distinct changes in the extracellular matrix that generate the mechanical cues to activate YAP/TAZ signaling in the epithelium (Yui et al., 2018). Strikingly, despite the widely reported observation of EGF stimulating proliferation of intestinal cells, there is little change in the expression of EGF during the regenerative response following injury in the intestinal epithelium (Jardé et al., 2020; Figure 2). In contrast, NRG1 is robustly up-regulated following injury in macrophages, endothelial cells and in PDGFRα+ stromal cells (Figure 2). The effect of NRG1 on the epithelium induces both a strong proliferative response and induction of stem cell characteristics in regenerating crypts (Jardé et al., 2020). AREG and EREG have also been observed to be induced in epithelial cells following injury, with knockout animals displaying a significant decrease in the number of regenerating crypt domains and a more significant weight loss following injury, respectively (Lee et al., 2004; Shao and Sheng, 2010; Table 1). The expression of ErbB3 is up-regulated during regeneration (Jardé et al., 2020) and the requirement for ErbB receptor function during tissue regeneration has been clearly demonstrated using knockout models. Indeed, loss of epithelial ErbB2 or ErbB3 decreases the ability of the intestine to efficiently regenerate following DSS-mediated injury (Lee et al., 2009; Zhang et al., 2012; Almohazey et al., 2017; Table 1).

## Utilizing Organoid Cultures to Interrogate Contributions of Ligands and Differential Activation of Signaling Pathways

It is clear that coordination of stem cell maintenance, progenitor proliferation and differentiation of mature cell types in the intestinal epithelium is orchestrated by gradients of active growth factors, agonists and antagonists *in vivo* (Tian et al., 2015; Basak et al., 2017; McCarthy et al., 2020b; Beumer and Clevers, 2021). The behavior of cells can change depending on the threshold and length of active signals and crosstalk between signaling pathways. Intestinal organoid culture was developed based on substitution of key *in vivo* niche signals (Sato et al., 2009, 2011) with Matrigel providing the appropriate stiffness to mimic the extracellular matrix. Under these conditions, the epithelium has the capacity to self organize, with many different epithelial cell types forming from single stem cells (Sato et al., 2009). Although this system has clear limitations, it provides an opportunity to study the intestinal epithelial population in isolation. Complex cellular interactions can be replicated by performing co-cultures with fibroblasts, nerve cells and immune cells (Kabiri et al., 2014; Rogoz et al., 2015; Pastula et al., 2016), in which genetic deletion of specific ligands can be performed and the impact on epithelial cells characterized. The effect of the microbiome and its by-products can also be evaluated (Mileto et al., 2020). However, and as opposed to applying the ligand-mediated stimulation in an unspecific manner in the current organoid technology, new systems that fully replicate the growth factor gradients observed *in vivo*, including WNT and EGF enrichment at the bottom of intestinal crypts, are required. Indeed, recent technological advances have allowed preservation of such complex tissue systems and gradients in culture using intestine- and organoid-on-a-chip models (Wang et al., 2018; Nikolaev et al., 2020). These systems will expand our understanding of complexity of the cellular microenvironment in which the cellular gradients of EGF family of ligands can be tested and assessed.

The relative proportion of different epithelial cell types can be manipulated in organoids by altering the culture conditions. For example, addition of CHIR99021 to elevate WNT signaling can enrich for Lgr5+ stem cells and inhibition of Notch induces secretory cells (Yin et al., 2014). Therefore, the organoid culture system permits the function of different environmental signals and signaling components to be interrogated by addition of proteins, toxins and chemical inhibitors (Clevers and Tuveson, 2019; Hageman et al., 2020). The function of the EGF family of ligands and receptors have been investigated using this methodology. EGF was included in the medium utilized for the first intestinal organoid cultures based on observations that EGF could promote proliferation of intestinal epithelial cells (McKenna et al., 1994; Dignass and Sturm, 2001; Abud et al., 2005; Sato et al., 2009). Along with R-spondin 1, EGF is required to maintain organoid crypt growth. Inhibition of signaling through the EGFR by addition of gefitinib and/or withdrawal of EGF from mouse small intestinal organoid cultures dramatically reduces proliferation within organoids and induces quiescence and an enteroendocrine molecular signature in Lgr5+ cells (Basak et al., 2017). However, one of the limitations in studying EGF signaling in intestinal organoids is the production by niche epithelial cells of EGF itself, which makes the analysis of exogenous supplementation vs. endogenous challenging.

It is important to keep in mind that the composition of the extracellular matrix surrounding epithelial cells in adult tissues, which is not fully replicated in organoid culture, might also affect the downstream molecular response to EGFR/ErbB activation (Yarwood and Woodgett, 2001). Other ligands from the family have also been tested in organoids. NRG1 can substitutes for EGF and robustly induces proliferation and budding of mouse small intestinal organoids through prolonged activation of MAPK and AKT signaling that is dramatically more effective than EGF (Jardé et al., 2020). In addition, HB-EGF supports the growth of human adult intestinal organoids in a similar fashion to EGF, which contrasts with the decreased ability of EREG to sustain organoid growth (Fujii et al., 2018). Human fetal enteroid cultures established in either NRG1 or EGF also display different phenotypes. EGF promotes proliferation and intestinal lineage identity while NRG1 supports cellular diversity and intestinal epithelial stem cell maturation (Holloway et al., 2021; Yu et al., 2021).

## Conclusion

A striking feature of the intestinal epithelium is the high turnover of cells which occurs on a daily basis, the dramatic expansion of tissue during embryonic development and the rapid remodeling observed in response to injury. Members of the EGF family of ligands and their receptors contribute substantially to these processes where the current evidence suggests there are distinct functions for particular ligands but also some functional redundancy (Gregorieff et al., 2015; Jardé et al., 2020; Holloway et al., 2021). The downstream signaling pathways that mediate these processes and the crosstalk that may occur with other pathways are less defined. Lineage tracing studies have revealed the high level of plasticity present within intestinal crypts with progeny of Lgr5+ cells having the capacity to de-differentiate following injury to replace the stem cell pool (Tian et al., 2011; Metcalfe et al., 2014; Schmitt et al., 2018; Yu et al., 2018; Ayyaz et al., 2019; Jones et al., 2019; Murata et al., 2020). Although several of the intrinsic signals required for this process have been identified, the influence of signals in the microenvironment that control this are less clear. It is likely that ligands such as NRG1, which is significantly upregulated in stromal cells during regeneration, play a role (Jardé et al., 2020). Organoid cultures offer a resource to further define these activities, especially for human tissues, and future studies incorporating co-cultures of specific niche cell types will further clarify cellular mechanisms. Defining these signals could ultimately inform strategies to improve epithelial repair in conditions such as inflammatory bowel disease, necrotizing enterocolitis and short gut syndrome.

## Author Contributions

HA and TJ wrote the manuscript. WC prepared the figures. HA, TJ, and WC edited and approved the final manuscript. All authors contributed to the article and approved the submitted version.

## Conflict of Interest

The authors declare that the research was conducted in the absence of any commercial or financial relationships that could be construed as a potential conflict of interest.
